# ENaC and Tight Junction Proteins: Coordinated Regulation of Alveolar Fluid Homeostasis in ALI/ARDS

**DOI:** 10.3390/ph19101642

**Published:** 2026-10-18

**Authors:** Shenting Li, Jinpeng Wang, Zhenhuan Yin, Shuning Xin, Hongguang Nie, Tong Yu

**Affiliations:** 1 Department of Stem Cells and Regenerative Medicine, College of Basic Medical Science, China Medical University, Shenyang 110122, China; lishenting@cmu.edu.cn (S.L.); wangjinpeng@cmu.edu.cn (J.W.); 2023110014@cmu.edu.cn (Z.Y.); xinshuning@cmu.edu.cn (S.X.); 2 Department of Urology, The Fourth Affiliated Hospital of China Medical University, Shenyang 110032, China; 3 Liaoning Key Laboratory of Bladder Disease Gene Research, Institute of Health Science, China Medical University, Shenyang 110032, China

**Keywords:** acute lung injury/acute respiratory distress syndrome, epithelial sodium channel, tight junction, pulmonary barrier function, alveolar fluid homeostasis

## Abstract

Acute lung injury (ALI) and its more severe form, acute respiratory distress syndrome (ARDS), remain critical clinical challenges characterized by acute diffuse lung inflammation, alveolar-capillary barrier disruption, and refractory pulmonary edema, leading to high morbidity and mortality. The alveolar epithelium, a key structural and functional unit of the lung, plays a pivotal role in maintaining alveolar fluid homeostasis, which depends on coordinated transcellular ion transport and paracellular barrier integrity mediated primarily by the epithelial sodium channel (ENaC) and tight junction proteins. As a central regulator of alveolar fluid clearance, ENaC mediates sodium ion reabsorption across the alveolar epithelium, whereas tight junction proteins, including zonula occludens-1, occludin, and claudins, restrict paracellular leakage and maintain epithelial barrier integrity. In the pathological setting of ALI/ARDS, both ENaC and tight junction proteins undergo significant dysregulation, with reduced ENaC function leading to impaired alveolar fluid clearance and exacerbated edema, while alterations in the expression, distribution, and function of tight junction proteins further compromise barrier integrity. Despite their individual roles being increasingly recognized, the complex interplay between ENaC and tight junctions in the alveolar fluid homeostasis of ALI/ARDS, as well as the underlying regulatory mechanisms, remain incompletely understood. This review therefore focuses on the mechanistic and functional crosstalk between ENaC and tight junction proteins, integrating their coordinated roles in alveolar fluid transport, barrier dysfunction, and pulmonary edema, and discussing potential therapeutic strategies with this coupled regulatory system in ALI/ARDS.

## 1. Introduction

Acute lung injury (ALI) or its more severe clinical manifestation, acute respiratory distress syndrome (ARDS), is an acute inflammatory lung disease that causes high rates of morbidity and mortality annually [1]. ALI/ARDS can result from a wide variety of direct lung injuries, such as pneumonia, or indirect insults such as sepsis [2]. Alveolar cells maintain the stability of alveolar structure, and disruption of alveolar-capillary barrier integrity or activation of inflammatory responses can cause dysfunctions in alveolar cells, leading to the accumulation of proteinaceous edema and inflammatory cells in the alveolar space, eventually progressing to ALI/ARDS [3]. The epithelial sodium channel (ENaC), an integral membrane protein, plays a crucial role in sodium reabsorption across various epithelial tissues [4]. It is primarily composed of three homologous subunits, α, β, and γ, which assemble to form a functional channel complex responsible for the selective transport of sodium ions across epithelial cell membranes [5]. Tight junction proteins, such as zonula occludens-1 (ZO-1), occludin, and claudins, are essential for maintaining the barrier function of alveolar epithelial cells [6].

To provide a transparent overview of the literature selection process, relevant studies were identified primarily through searches of PubMed, Web of Science, and Google Scholar. Search terms included combinations of “acute lung injury”, “acute respiratory distress syndrome”, “epithelial sodium channel”, “ENaC”, “alveolar fluid clearance”, “tight junction”, “ZO-1”, “occludin”, and “claudin”. The literature search focused primarily on studies published from 2018 to 2026, while earlier landmark studies were also included when they provided important mechanistic or foundational evidence. Priority was given to peer-reviewed original studies and reviews directly addressing ENaC-mediated ion transport, alveolar epithelial barrier function, tight junction regulation, and their relevance to ALI/ARDS.

ENaC-mediated transcellular sodium transport promotes alveolar fluid clearance (AFC), whereas tight junction proteins regulate paracellular permeability and restrict ion back-leak, representing two complementary components of alveolar fluid homeostasis [7]. Thus, effective alveolar fluid homeostasis depends not on either pathway alone, but on the coordinated balance between transcellular fluid clearance and paracellular barrier integrity. In the context of ALI/ARDS, dysregulation of either system may influence the other, thereby amplifying epithelial barrier dysfunction and impairing fluid clearance. These reciprocal effects provide a mechanistic basis for considering ENaC and tight junction proteins as an interconnected regulatory system rather than as independent contributors to lung injury [8].

## 2. Disturbed Alveolar Fluid Homeostasis in ALI/ARDS

### 2.1. Pathogenic Causes of ALI/ARDS

The etiology of ALI/ARDS is complex and varied, the main direct causes of which are severe pneumonia, aspiration of gastric contents, pulmonary contusion, and inhalation of toxic gases [9]. For example, in severe pneumonia, the pathogen and its toxins directly damage the alveolar epithelium and pulmonary vascular endothelial cells, triggering an inflammatory response that leads to ALI/ARDS [10]. Aspiration of gastric contents can cause chemical inflammation that disrupts the alveolar epithelial barrier, activates inflammatory cells, and releases inflammatory mediators. Moreover, extrapulmonary indirect injury factors are common in severe trauma, sepsis, shock, and massive blood transfusions. In sepsis, endotoxins released by bacteria can activate the systemic inflammatory response; produce a large number of cytokines and inflammatory mediators, which reach the lungs through the blood circulation, then damage the alveolar epithelium and pulmonary vascular endothelial cells; and cause ALI/ARDS [11]. In mechanically ventilated or stretch-based experimental models, alveolar epithelial cells are exposed to cyclic mechanical forces that may alter cytoskeletal tension and tight junction organization [12]. These etiological differences are mechanistically relevant because direct epithelial injury, systemic inflammatory injury, mechanical stress, and hypoxic/oxidative injury do not necessarily produce identical effects on ENaC expression, membrane localization, tight junction organization, or barrier permeability [10,13]. Accordingly, observations obtained in one ALI/ARDS model should not automatically be generalized to all forms of lung injury [2]. In severe systemic or pulmonary inflammation, amplification of cytokine and chemokine release may further recruit and activate neutrophils and other inflammatory cells, thereby intensifying oxidative stress and alveolar-capillary barrier injury [14]. Experimental lipopolysaccharide (LPS)-induced cytokine-storm models support this inflammatory amplification as an important contributor to lung injury [15,16].

### 2.2. Impaired AFC in ALI/ARDS

During ALI/ARDS, AFC is significantly reduced. ENaC located at the apical membrane mediates Na^+^ entry from the alveolar lumen into epithelial cells, whereas basolateral Na^+^/K^+^-ATPase extrudes intracellular Na^+^ toward the interstitial side and thereby maintains the electrochemical gradient required for continued apical Na^+^ entry [8]. Thus, AFC reflects the integrated balance between transcellular Na^+^ absorption, basolateral Na^+^ extrusion, paracellular permeability, and accompanying water movement [17]. Other epithelial ion-transport pathways that contribute to membrane potential and electrochemical coupling may further modulate this process. In addition, damage to alveolar epithelial cells, leading to cellular dysfunction, is also detrimental to AFC [18]. The formation of pulmonary edema further exacerbates accumulation of fluid in the alveoli, creating a vicious cycle that leads to disease progression.

### 2.3. Disruption of Alveolar Epithelial Barrier Function in ALI/ARDS

During ALI/ARDS, alveolar epithelial barrier function is severely disrupted, with structural and functional alterations of intercellular tight junctions representing an important component of epithelial injury [19,20]. Inflammatory mediators and oxidative stress can disrupt the organization of tight junction complexes and alter the expression and localization of key junctional proteins, thereby weakening epithelial barrier integrity [21,22]. Of note, the expression levels of tight junction proteins such as ZO-1 and occludin are reduced and these proteins can redistribute from the intercellular junction to the cytoplasm, resulting in discontinuous junctional organization, widened intercellular spaces and increased permeability of the alveolar epithelium [19]. Consequently, protein-rich fluid and other plasma components can cross the compromised alveolar epithelial barrier and accumulate within the alveolar space, thereby promoting pulmonary edema and impairing gas exchange [23,24]. At the same time, damage to alveolar epithelial cells can also lead to increased apoptosis, further disrupting the integrity of the alveolar epithelial barrier [25].

## 3. Reduced ENaC Function Leading to Impaired AFC

ENaC is a member of the non-voltage-gated ion channel family, mainly composed of α, β, and γ subunits. In humans, there is an additional δ subunit, which is expressed at relatively low levels [26]. The β and γ subunits interact with α subunit to regulate ion selectivity and channel opening probability, while also contributing to the channel’s stability and functionality [27]. For instance, when the α, β, and γ subunits assemble together to form a heteropolymer, the channel exhibits a high selectivity for sodium ions, efficiently mediating the transmembrane transport of sodium ion [28]. There is a close relationship between the structure and function of ENaC. Its unique subunit composition and transmembrane structure confer high ionic selectivity to the channel, allowing it to specifically recognize and transport sodium ion, while having lower permeability to other ions such as potassium and calcium [29]. In addition, the opening and closing of the channel are finely regulated by various factors, including hormones, neurotransmitters, intracellular signaling pathways, and membrane potential [30]. Through binding to intracellular receptors, aldosterone hormone can stimulate the cells, which leads to increased synthesis of ENaC subunits, enhanced membrane expression of the channel, and higher probability of channel opening, thereby enhancing sodium ion transport [29,31].

In terms of ion transport characteristics, ENaC has a high sodium ion selectivity, with its permeability to sodium ion being much higher than that of other cations [32]. At the same time, ENaC has a relatively low transport rate, classifying it as a low-flux ion channel, yet its precision and stability in transport ensure the body’s precise regulation of sodium ion homeostasis [5]. In alveolar epithelial cells, ENaC maintains fluid balance within the alveoli by continuously and stably transporting sodium ions, providing a favorable microenvironment for gas exchange.

### 3.1. Abnormal ENaC Function Disrupting Alveolar Fluid Transport

ENaC is expressed on the apical membrane of alveolar epithelial cells and mediates sodium entry from the alveolar lumen into epithelial cells, which constitutes a major driving force for AFC and is essential for maintaining alveolar fluid homeostasis. Specifically, Na^+^ enters alveolar epithelial cells from the alveolar lumen through ENaC on the apical membrane, thereby initiating Na^+^ absorption [33]. Subsequently, the Na^+^/K^+^-ATPase located on the basolateral membrane utilizes energy generated by ATP hydrolysis to transport Na^+^ from epithelial cells towards the interstitial/capillary side [34]. This vectorial sodium transport establishes an osmotic gradient across the alveolar epithelium, which drives the passive movement of water from the alveolar space towards the interstitium and circulation [35,36]. Importantly, the magnitude of net transepithelial Na^+^ transport depends not only on transcellular entry through ENaC and basolateral extrusion by Na^+^/K^+^-ATPase, but also on the ability of tight junctions to restrict paracellular Na^+^ back-leak [7]. An intact tight junction barrier helps maintain the transepithelial electrochemical gradient generated by active Na^+^ transport. When tight junctions become disrupted, increased paracellular conductance allows absorbed Na^+^ to move back toward the alveolar lumen, thereby dissipating the transepithelial gradient and reducing net Na^+^ absorption. Consequently, the osmotic force driving alveolar fluid removal is weakened even if ENaC remains partially active [37].

During the progression of ALI/ARDS, abnormal ENaC function can therefore directly impair AFC and contribute to pulmonary edema. However, the extent and mechanism of ENaC dysfunction are context-dependent. In endotoxin or cytokine dominant models, transcriptional suppression and inflammatory signaling may predominate, whereas oxidative stress can additionally alter post-translational regulation and membrane abundance [13,38]. Hypoxia may influence ENaC through redox-sensitive and metabolic mechanisms, while mechanical injury may alter membrane trafficking, cytoskeletal organization, and epithelial polarity. ENaC regulation occurs at several distinct but interconnected levels. First, transcriptional and translational regulation determine the total cellular abundance of ENaC subunits [39]. Second, trafficking, membrane insertion, ubiquitination, internalization, and degradation determine the amount of ENaC present at the apical membrane [40]. As an E3 ubiquitin ligase, NEDD4-2 promotes ubiquitination of ENaC subunits, which favors channel internalization and subsequent removal from the apical membrane [38]. In the setting of oxidative or inflammatory stress, dysregulation of this pathway may contribute to reduced ENaC-mediated Na^+^ absorption and impaired AFC [41]. Third, proteolytic processing and other post-translational mechanisms regulate the activity and open probability of membrane-resident channels [30,40]. During channel maturation, furin cleaves extracellular regions of the α- and γ-subunits. Subsequent cleavage of γ-ENaC by extracellular serine proteases, including prostasin and neutrophil elastase, can remove inhibitory extracellular segments and increase channel open probability. Importantly, this mechanism primarily modifies the activity of channels already present at or delivered to the plasma membrane and should therefore be distinguished from mechanisms controlling ENaC transcription, total protein expression, or membrane trafficking [26].

**Figure 1:**
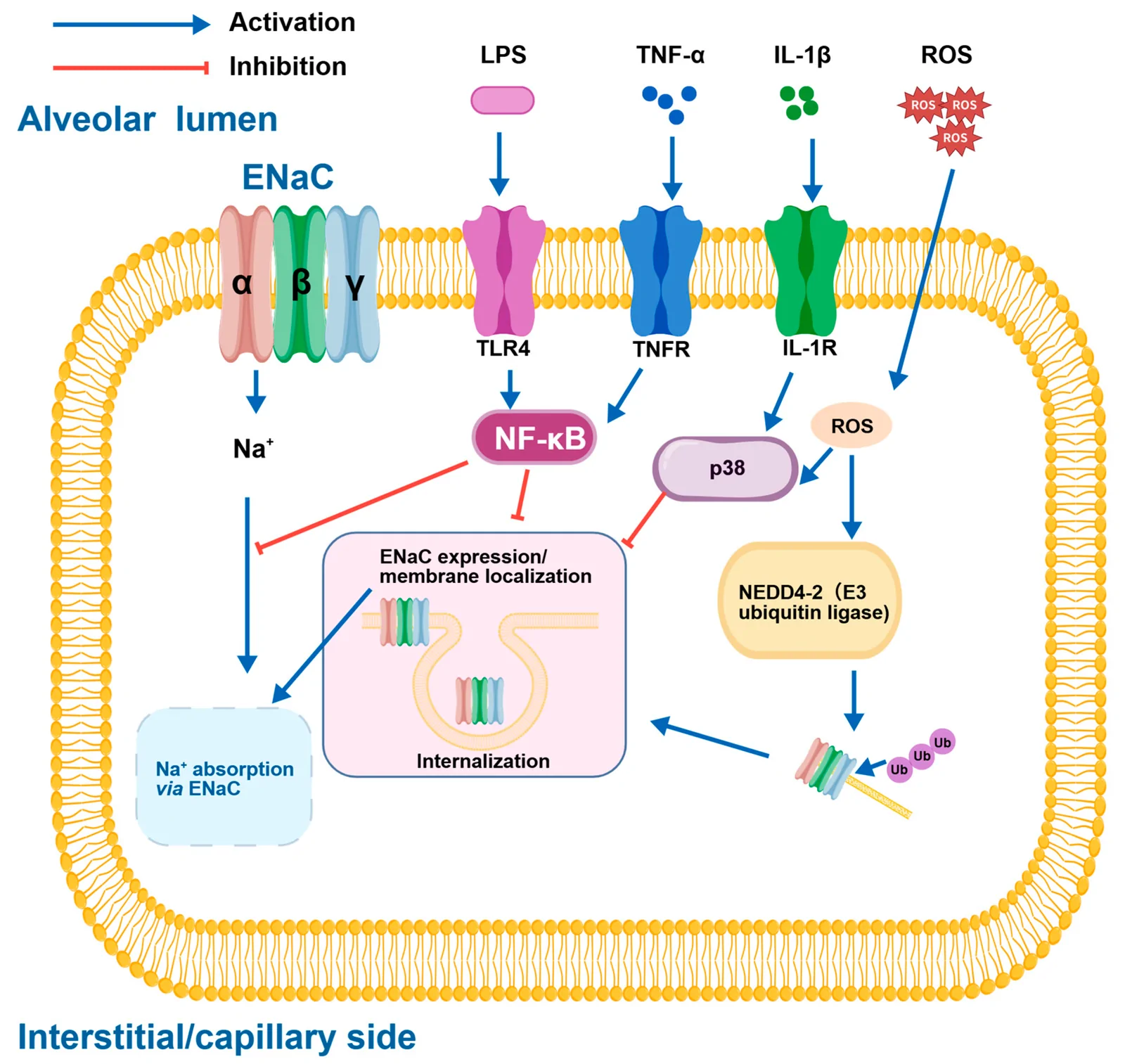
In the pathogenesis of ALI/ARDS, inflammatory and oxidative stimuli disrupt ENaC-mediated sodium transport through interconnected signaling pathways. Inflammatory signaling mediated by NF-κB and p38, together with ROS-associated NEDD4-2 regulation, reduces ENaC expression, membrane localization, and channel activity, ultimately impairing epithelial Na^+^ transport and alveolar fluid clearance. Blue arrows indicate activation or stimulatory effects, whereas red blunt-ended lines indicate inhibitory effects. ALI, acute lung injury; ARDS, acute respiratory distress syndrome; ENaC, epithelial sodium channel; LPS, lipopolysaccharide; IL-1β, interleukin-1β; IL-1R, interleukin-1 receptor; NEDD4-2, neural precursor cell expressed developmentally downregulated protein 4-2; NF-κB, nuclear factor kappa B; p38, p38 mitogen-activated protein kinase; TLR4, toll-like receptor 4; ROS, reactive oxygen species; TNF-α, tumor necrosis factor-α; TNFR, tumor necrosis factor receptor; Ub, ubiquitin. The figure was created at https://BioGDP.com (accessed on 6 September 2026).

In ALI/ARDS, inflammatory mediators, oxidative stress, and mechanical injury may disturb one or more of these regulatory levels, ultimately reducing ENaC-mediated sodium transport (Figure 1) [40]. Consequently, sodium uptake by alveolar epithelial cells is reduced, weakening the osmotic gradient required for water reabsorption [42]. Impaired fluid clearance promotes fluid accumulation within the alveolar space and aggravates pulmonary edema [43]. The resulting disruption of gas exchange can further contribute to hypoxemia and exacerbate lung injury [44]. Inflammatory and oxidative stimuli are important factors contributing to ENaC dysfunction during ALI/ARDS. An earlier study using A549 lung epithelial cell line demonstrated that hydrogen peroxide inhibited dexamethasone-induced activation of α-ENaC transcription [45], suggesting that oxidative stress can interfere with ENaC expression and epithelial sodium transport. Oxidative stress/reactive oxygen species (ROS) commonly accompanies pulmonary infection and inflammation, and thus alter epithelial ion transport mechanisms, thereby impairing alveolar fluid removal and potentially delaying recovery from lung injury [46]. In parallel, inflammatory mediators can suppress ENaC expression and function [47]. For example, LPS activates inflammatory signaling pathways and promotes the production of TNF-α, interleukin (IL)-1β, and other inflammatory mediators, which further inhibit ENaC-mediated sodium transport and contribute to impaired AFC [13].

### 3.2. Moderators of ENaC Function

The functional output of ENaC reflects the combined effects of gene expression, synthesis of channel subunits, delivery to and retention at the apical membrane, proteolytic activation, channel gating, and removal through ubiquitination-dependent internalization and degradation [26]. These regulatory processes are highly interconnected. Therefore, factors that alter ENaC-mediated sodium transport may act at transcriptional, trafficking, post-translational levels, or at several levels simultaneously [30].

#### 3.2.1. Hormone Regulation

Aldosterone is an important hormonal regulator of ENaC function [48]. After binding to mineralocorticoid receptors in type II alveolar epithelial cells, aldosterone activates ENaC-related gene transcription, promotes the synthesis of ENaC subunits, and facilitates their trafficking to the apical membrane, thereby enhancing ENaC activity and promoting sodium and fluid reabsorption. In parallel, aldosterone-dependent signaling facilitates ENaC trafficking and retention at the apical membrane, and increasing membrane channel abundance. These combined changes can enhance ENaC-mediated sodium transport and promote alveolar fluid reabsorption [49]. Angiotensin II can also regulate ENaC through angiotensin II type 1 receptor-dependent signaling [50]. In addition to stimulating aldosterone secretion and thereby indirectly regulating ENaC, angiotensin II can directly act on type II alveolar epithelial cells through angiotensin II type 1 receptor-dependent signaling, which has been shown to reduce intracellular cAMP levels, dysregulate ENaC expression, and inhibit AFC [8].

#### 3.2.2. Cytokines and Inflammatory Mediators

Excessive inflammatory activation is a central feature of many forms of ALI/ARDS [14]. Following infectious or sterile injury, activated immune and structural cells can release high levels of pro-inflammatory mediators, including TNF-α, IL-1β, IL-6, chemokines, and other signaling molecules [2]. When this response becomes excessive, a cytokine-amplification or “cytokine storm”-like state may promote neutrophil recruitment, oxidative stress, epithelial and endothelial injury, and increased alveolar-capillary permeability [13]. Experimental LPS-induced lung-injury and cytokine-storm models further support the contribution of exaggerated inflammatory mediator release to pulmonary tissue injury and barrier dysfunction [15,16]. In inflammatory states, a variety of cytokines and inflammatory mediators exert significant effects on ENaC. TNF-α and IL-1β can inhibit the expression and channel activity of ENaC subunits [51], and these effects may be associated with activation of mitogen-activated protein kinase and NF-κB signaling pathways, which in turn influence ENaC gene transcription, ENaC expression/membrane localization, and post-translational regulation. LPS activates toll-like receptor 4-dependent NF-κB signaling, whereas TNF-α acts through the TNF receptor and IL-1β acts through the IL-1 receptor to activate downstream NF-κB and/or p38 MAPK signaling [39]. These observations place ENaC dysregulation within a broader inflammatory network in which excessive cytokine signaling, neutrophilic inflammation, and oxidative stress can simultaneously impair epithelial ion transport and barrier integrity [9,39].

#### 3.2.3. Other Moderating Factors

Other modulators of ENaC during ALI/ARDS can be broadly grouped into oxidative, cytoskeletal, and neurohumoral factors. The partial pressure of oxygen also modulates ENaC activity. Hypoxia inhibits ENaC activity and reduces sodium ion transport, possibly through increased production of ROS [41,52,53], thereby activating redox-sensitive signaling pathways and affecting ENaC regulation. In addition, cytoskeletal proteins such as actin can interact with ENaC and regulate its membrane abundance and channel activity [54]. Changes in cytoskeletal organization may therefore influence ENaC trafficking and membrane stability, providing a potential link between mechanical stress and epithelial sodium transport [55]. Among neurohumoral factors, β-Adrenergic stimulation can enhance ENaC activity and promote AFC through receptor-dependent signaling pathways [56].

## 4. Alterations of Tight Junction Proteins Compromising Barrier Integrity

The alveolar-capillary barrier consists of two closely apposed but functionally distinct compartments: the alveolar epithelial barrier and the pulmonary microvascular endothelial barrier. The epithelial compartment, formed mainly by alveolar epithelial cells and their tight junctions, limits paracellular leakage into the alveolar space and participates directly in ion and fluid transport [57]. In contrast, the endothelial compartment regulates vascular permeability and the movement of plasma fluid and proteins from the pulmonary circulation into the interstitial space [58]. Dysfunction of either compartment can contribute to pulmonary edema, whereas simultaneous epithelial and endothelial injury markedly amplifies alveolar-capillary barrier failure in ALI/ARDS [20]. In alveolar epithelial cells, ZO-1 is arranged continuously along the edges of the cells, forming a closed circular structure that tightly connects adjacent cells together [59]. This localization enables it to effectively regulate paracellular permeability, preventing large molecules and pathogens from entering the tissues through intercellular spaces [60]. In ALI/ARDS, ZO-1 undergoes Ser/Thr phosphorylation via TNF-α/IL-1β-activated Ras homolog family member A/Rho-associated coiled-coil-containing protein kinase (RhoA/ROCK) pathway [61], leading to its translocation from the cell membrane to the cytoplasm. This change not only precedes the degradation of claudins/occluding, but also directly destroys the overall structure of tight junctions.

In contrast, claudins mainly affect ion-selective permeability, while occludin is more susceptible to MMP-mediated degradation, thereby impairing macromolecular paracellular permeability [62]. It should be noted that some tight junction mechanisms described in ALI/ARDS primarily involve the pulmonary vascular endothelium rather than the alveolar epithelium. ZO-1, occludin, and epithelial claudins are considered mainly in the context of alveolar epithelial barrier integrity, whereas claudin-5 and some junction adhesion molecule-A (JAM-A)-related mechanisms are identified explicitly as endothelial when the supporting evidence concerns vascular permeability. In endothelial cells, JAM-A-dependent signaling can promote claudin-5 expression and thereby support endothelial barrier integrity [58].

### 4.1. Maintaining Tight Junctions and Barrier Function in Normal Alveolar Epithelial Cells

Among these tight junction proteins, ZO-1 plays a central role in maintaining tight junction organization by linking transmembrane proteins to the actin cytoskeleton [63,64,65]. ZO-1 contains three postsynaptic density-95/discs-large/zonula occludens-1 (PDZ) domains, an Src homology 3 domain, and a guanylate kinase domain [66]. These structural domains enable ZO-1 to interact with various tight junction components, including claudins and occludin, and mediate protein–protein interactions, thereby regulating tight junction assembly, stability, and barrier function [67,68]. When the expression or function of ZO-1 is inhibited, the integrity of tight junctions is compromised, and paracellular permeability significantly increases. In in vitro cell experiments, reducing the expression of ZO-1 using RNA interference technology resulted in a marked decrease in the transepithelial electrical resistance of the alveolar epithelial cell monolayer [65], along with a significant increase in the permeability of fluorescein-labeled dextran, indicating impaired paracellular barrier function. When cells are stimulated by external factors, ZO-1 can sense and transmit signals to relevant signaling pathways within the cell, triggering adaptive responses [69]. During lung injury repair, ZO-1 promotes the repair and regeneration of alveolar epithelial cells by regulating cell migration and proliferation, thereby maintaining the integrity of the pulmonary epithelial barrier [70]. Furthermore, ZO-1’s interaction with the cytoskeleton is also crucial for maintaining the morphology and barrier function of normal alveolar epithelial cells [71]. By connecting with actin filaments, it integrates tight junctions with the cytoskeletal network, providing structural support to the cells. This interaction not only helps maintain normal cell morphology but also regulates cellular mechanical stability, enabling them to resist external forces [72].

Occludin is another important transmembrane component of tight junctions and contributes to the stabilization of intercellular junctions and the regulation of paracellular permeability [73]. Alterations in the expression or localization of these proteins can disrupt tight junction organization and increase epithelial permeability, thereby promoting alveolar barrier dysfunction. Claudins constitute major transmembrane components of tight junctions and play a central role in determining paracellular ion selectivity and permeability. Among alveolar epithelial claudins, claudin-18 is highly enriched in the lung epithelium and contributes to the low paracellular permeability characteristic of the alveolar barrier. By restricting cation movement through the paracellular pathway, claudin-18 helps preserve transepithelial ionic gradients required for efficient vectorial ion transport. Claudin-4 also functions as a barrier-forming claudin in the alveolar epithelium and is associated with increased transepithelial resistance and reduced paracellular permeability [74]. Its upregulation during lung injury has been linked to restoration of epithelial barrier function and improved fluid clearance. Thus, claudin-18 contributes predominantly to baseline alveolar barrier selectivity, whereas claudin-4 is particularly relevant to barrier reinforcement and repair during injury [37,75].

### 4.2. Changes in Tight Junction Proteins in ALI/ARDS

Reduced expression and abnormal redistribution of key tight junction proteins have been reported in various experimental models of ALI/ARDS and in patients [76]. These changes are also stage dependent. During the acute injury phase, inflammatory mediators, oxidative stress, and mechanical disruption can promote loss or redistribution of junctional proteins and increase paracellular permeability [76]. During the subsequent repair phase, reassembly of ZO-1, occludin, and barrier-forming claudins may accompany epithelial restitution, repolarization, and restoration of barrier function [37,65]. Therefore, reduced junctional abundance during acute injury and increased junctional reassembly during repair represent different phases of the epithelial response rather than contradictory findings. During the process of ALI/ARDS, ZO-1 undergoes significant changes. Factors such as inflammatory mediators and oxidative stress can lead to downregulation of ZO-1 expression, abnormal distribution, and weakened interactions with other proteins [21,22,77]. These changes in ZO-1 can lead to the disruption of tight junctions in alveolar epithelial cells, increasing paracellular permeability, and causing large amounts of proteins, fluids, and inflammatory cells to leak into the alveolar cavity and lung interstitium, triggering pulmonary edema and exacerbating the inflammatory response [23,24]. The formation of pulmonary edema will further affect gas exchange, leading to hypoxemia and carbon dioxide retention, worsening the condition. By regulating ZO-1 expression and function, it is possible to improve the tight junctions and barrier function of alveolar epithelial cells, alleviating pulmonary edema and the inflammatory response [78]. Experimental and preclinical studies suggest that statins and antioxidant strategies can modulate inflammatory or oxidative signaling and may preserve tight junction proteins such as ZO-1 in selected models [79]. Additionally, emerging technologies like gene therapy and cell therapy offer new avenues for modulating ZO-1 function [80].

Besides ZO-1, other tight junction proteins also undergo substantial alterations during ALI/ARDS [57]. Occludin expression is frequently reduced, accompanied by changes in its phosphorylation status and redistribution away from intercellular junctions, which weaken tight junction stability and increase paracellular permeability. In addition to reduced expression, occludin undergoes marked alterations in its junctional distribution during ARDS [66]. Recent evidence from human ARDS lung tissues demonstrated substantially decreased occludin expression together with loss of its normal linear junctional distribution, indicating disruption of the alveolar epithelial barrier. Experimental reduction in occludin further increased lung injury and barrier permeability, whereas restoration of occludin expression attenuated endotoxin-induced lung injury. Therefore, preservation of occludin abundance and junctional localization may represent a potential strategy for maintaining alveolar epithelial barrier integrity in ARDS.

Claudins also exhibit subtype-dependent alterations in expression and localization. Inflammatory stimuli such as LPS and IL-1β, together with matrix metalloproteinase activity, can disrupt claudin expression and organization, thereby impairing the ion-selective barrier of the alveolar epithelium [37]. Moreover, oxidative stress and altered epithelial signaling may contribute to claudin dysregulation. Accordingly, restoration of barrier-forming claudins and stabilization of paracellular ion permeability may represent potential strategies for improving alveolar epithelial barrier function [81]. Moreover, JAM-A may undergo proteolytic degradation during excessive inflammatory responses, further compromising junctional integrity and facilitating inflammatory cell migration [82]. During inflammatory or mechanical injury, reduced junctional abundance and proteolytic shedding of JAM-A may further compromise junctional integrity. ADAM10- and ADAM17-mediated shedding has been implicated in the loss of surface JAM-A under inflammatory conditions [83]. The major functions, pathological alterations, regulatory factors, and potential therapeutic targets of tight junction proteins in ALI/ARDS are summarized in Table 1.

## 5. Interrelationship Between ENaC and Tight Junction Proteins in ALI/ARDS

**Figure 2:**
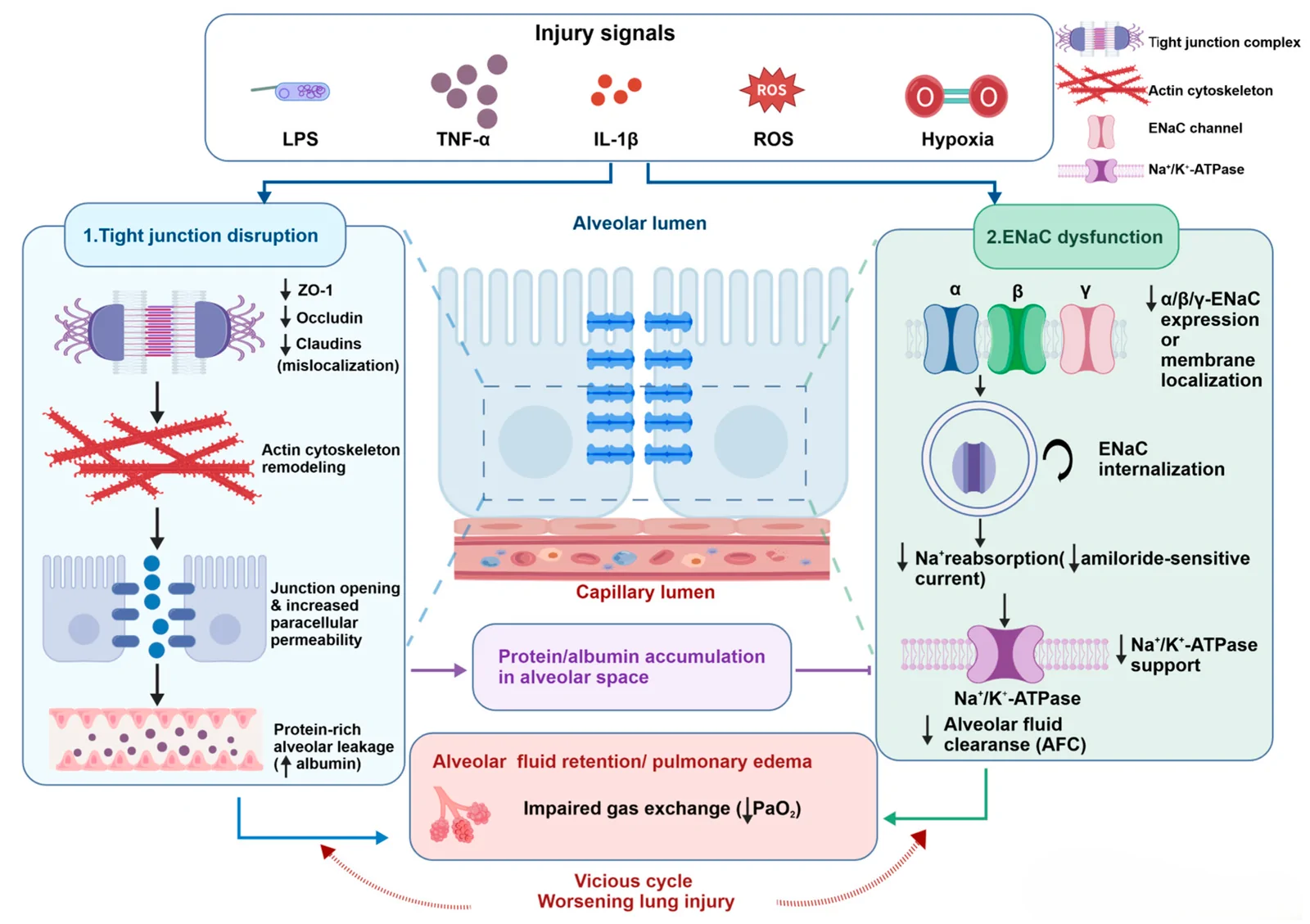
In the pathogenesis of ALI/ARDS, inflammatory, oxidative, and hypoxic injury signals coordinately impair alveolar epithelial barrier integrity and ENaC-mediated sodium transport, thereby disrupting pulmonary fluid homeostasis. Disruption of ZO-1, occludin, and claudins increases paracellular permeability and protein-rich fluid leakage, whereas reduced ENaC expression and membrane localization, together with impaired Na^+^/K^+^-ATPase function, decreased transcellular Na^+^ transport and AFC. The resulting alveolar fluid retention and pulmonary edema further compromise gas exchange and amplify lung injury, forming a self-reinforcing vicious cycle in ALI/ARDS. AFC, alveolar fluid clearance; ALI, acute lung injury; ARDS, acute respiratory distress syndrome; ENaC, epithelial sodium channel; ZO-1, zonula occludens-1. The figure was created at https://BioGDP.com (accessed on 6 September 2026).

ENaC and tight junction proteins are functionally interconnected in maintaining alveolar fluid homeostasis and epithelial barrier integrity [7]. As illustrated in Figure 2, multiple injury signals, including LPS, TNF-α, IL-1β, ROS, and hypoxia, can simultaneously disturb the epithelial barrier and alveolar ion transport, resulting in two closely coupled pathological processes, tight junction disruption and ENaC dysfunction. On the barrier side, disruption of the tight junction complex, involving altered expression or mislocalization of ZO-1, occludin, and claudins, together with actin cytoskeleton remodeling, promotes junction opening and increased paracellular permeability. Consequently, protein-rich alveolar leakage, particularly increased albumin leakage, occurs from the capillary lumen towards the alveolar lumen, resulting in protein/albumin accumulation in the alveolar space [84]. On the ion-transport side, ENaC dysfunction is characterized by decreased α/β/γ-ENaC expression or membrane localization, enhanced ENaC internalization, and impaired ENaC channel activity. These alterations reduce Na^+^ reabsorption, including the amiloride-sensitive current, while reduced Na^+^/K^+^-ATPase support further compromises vectorial sodium transport across the alveolar epithelium. The combined impairment of ENaC-mediated sodium transport and Na^+^/K^+^-ATPase activity ultimately decreases AFC [8]. Therefore, tight junction disruption increases fluid and protein entry into the alveolar space, whereas ENaC dysfunction limits their removal, jointly promoting alveolar fluid retention/pulmonary edema and impaired gas exchange, reflected by decreased PaO_2_. These mutually reinforcing abnormalities establish a vicious cycle, thereby disturbing alveolar fluid homeostasis in ALI/ARDS.

### 5.1. ZO-1 and ENaC: Pivotal and Indirect Regulation of Signaling Pathways

In alveolar epithelial cells, ENaC is primarily responsible for the transport of sodium ions, which drives the reabsorption of water molecules, thereby facilitating alveolar liquid clearance and maintaining fluid balance within the alveoli [85]. ZO-1, on the other hand, primarily participates in maintaining the integrity and stability of tight junctions between alveolar epithelial cells, regulating paracellular permeability, and preventing the passage of large molecules and pathogens, providing an effective physical barrier for the alveoli [65]. Compared with other tight junction proteins ZO-1, as a core scaffolding protein, connects transmembrane proteins like claudin-4, occludin to the actin cytoskeleton to maintain barrier integrity. The relationship between ZO-1 and ENaC in the alveolar epithelium is more appropriately regarded as functional crosstalk, involving coordinated regulation of epithelial barrier integrity, ion transport, cellular polarity, and the local ionic microenvironment, rather than as an established direct protein–protein interaction [78,86].

Evidence from experimental epithelial cell systems suggests that changes in junctional organization and Rho-dependent signaling may influence ENaC membrane abundance and channel activity [55]. Enhanced ENaC activity increases sodium transport and may alter intracellular ionic homeostasis and osmotic balance, which may affect the interactions of ZO-1 with other tight junction proteins and the actin cytoskeleton, thereby modulating tight junction organization and barrier function [87,88]. Under physiological hormonal stimulation, increased ENaC activity has been associated with more stable junctional localization of ZO-1 and reduced paracellular permeability. Conversely, when ENaC is dysfunctional, it may lead to an imbalance in intracellular ion homeostasis, thereby affecting the normal function of ZO-1, compromising the integrity of tight junctions and increasing paracellular permeability [89]. In pathological conditions such as ALI/ARDS, decreased expression and activity of ENaC can lead to abnormal distribution of ZO-1, changing from a normal continuous linear distribution to a discontinuous punctate distribution, impairing the barrier function of tight junctions and triggering pulmonary edema and inflammatory responses [7].

ZO-1 can also influence the expression and activity of ENaC by regulating cellular signaling pathways that can interact with various signaling molecules, which are involved in multiple biological processes within the cell, including the regulation of gene expression [84]. Additionally, in ALI/ARDS, functional crosstalk between ZO-1 and ENaC may contribute to the coordinated regulation of alveolar barrier integrity and fluid clearance [90]. Alterations in ENaC activity can influence membrane potential and intracellular ionic homeostasis, which in turn affect the phosphorylation, distribution, and stability of ZO-1 at intercellular junctions. In particular, excessive ENaC activation may induce apical membrane depolarization and promote tyrosine phosphorylation and redistribution of ZO-1, thereby disrupting tight junction organization and increasing paracellular permeability [91]. Thus, the interaction between ENaC-mediated sodium transport and ZO-1-dependent tight junction integrity represents an important mechanism linking AFC to epithelial barrier function. Some studies suggest that when cells are subjected to external stimuli, ZO-1 can influence the membrane expression and channel activity of ENaC by regulating the Rho signaling pathway, thus modulating the pulmonary fluid clearance function [55]. Additionally, ZO-1 can maintain cell morphology and structural stability by interacting with the cytoskeleton, providing a favorable intracellular environment for the normal function of ENaC. When the connection between ZO-1 and the cytoskeleton is disrupted, it impacts cell morphology and polarity, subsequently interfering with the localization and function of ENaC [55,92]. At the cytoskeletal level, ZO-1 bridges transmembrane junctional proteins with the actin network via its PDZ domains, forming the structural foundation of the epithelial barrier. During ALI/ARDS, inflammatory factors activate the RhoA/ROCK/MLC2 pathway, triggering dissociation of this linkage, which leads to compromised tight junction integrity, barrier dysfunction, and subsequent plasma albumin leakage into the alveolar space [93]. The accumulated albumin directly inhibits ENaC activity by binding to its pore-forming domains, establishing a vicious cycle between tight junction disruption and AFC impairment. At the signaling level, ZO-1 serves as a molecular platform recruiting key regulatory factors. Inflammation-activated protein kinase C (PKC) phosphorylates specific serine/threonine residues on ZO-1, causing its translocation from the membrane to the cytoplasm which accelerates tight junction disassembly and compromises ENaC function.

### 5.2. Occludin and ENaC: Cross-Talk Regulated by Phosphorylation

As a transmembrane protein regulating the dynamics of tight junctions, occludin can indirectly modulate the function of ENaC through its phosphorylation status, which affects barrier integrity and signaling pathways [94]. In terms of the indirect role in barrier stability, the C-terminus of occludin binds to ZO-1 to maintain tight junction sealing [95]. During ALI/ARDS, occludin undergoes internalization and degradation, which may increase paracellular permeability and thereby increase exposure of the alveolar surface to protein-rich fluid, potentially contributing indirectly to inhibition of ENaC function [96].

For tyrosine phosphorylation, inflammation activates Src to phosphorylate occludin at tyrosine residues, resulting in the dissociation of occludin from ZO-1, tight junction disintegration, and indirect inhibition of ENaC. For threonine phosphorylation, PKC phosphorylates occludin at Thr-404/403, promoting tight junction assembly, maintaining low alveolar luminal protein concentration, and thus protecting ENaC from inhibition. Experimental evidence shows that treatment of alveolar epithelial cells with a PKC inhibitor reduces ENaC activity. Co-damage caused by oxidative stress also exists. ROS oxidize cysteine residues of occludin, accelerating its ubiquitination and degradation. Meanwhile, ROS directly oxidize the α-subunit of ENaC, reducing the channel open probability. Degradation of occludin leads to tight junction leakage, increased alveolar luminal albumin concentration, while direct inhibition of ENaC function, reduced sodium ion absorption, and ultimately edema fluid retention, forms a vicious cycle of synergistic dysregulation [97].

### 5.3. Claudins and ENaC: Synergy and Competition for Ion Selectivity

As key transmembrane proteins determining the ion selectivity of tight junctions, claudins interact dynamically with ENaC-mediated transcellular transport pathways by regulating paracellular ion transport [93]. Among different claudin family members, the barrier-forming claudin-4 acts as an “indirect enhancer” of ENaC by restricting paracellular sodium back-leak, thereby maintaining transepithelial electrical resistance, stabilizing the negative potential on the alveolar luminal side, and enhancing ENaC-mediated transcellular sodium transport [98]. Claudin-4 does not directly activate ENaC, whereas its barrier-forming effect improves the efficiency of net transepithelial Na^+^ transport by limiting paracellular Na^+^ recycling [76]. In ALI/ARDS, however, downregulation of claudin-4 leads to increased sodium backflow, thereby reducing the efficiency of net transepithelial Na^+^ absorption [99]. Claudin-18, which is specifically expressed in alveoli, functions as a guardian of the cation barrier whose deficiency increases the permeability of tight junctions to sodium ions, leading to a decrease in transepithelial potential difference and thereby weakening the electrochemical driving force for net epithelial Na^+^ absorption [75]. During ALI/ARDS, disruption of claudin-18 and claudin-4 expression or localization may therefore increase paracellular Na^+^ permeability and reduce net transepithelial Na^+^ and fluid transport, thereby contributing to impaired AFC [94,100].

### 5.4. Potential Linkages of Other Tight Junction-Related Proteins to ENaC

Beyond core tight junction proteins, additional junction-associated molecules may establish pathological links with ENaC through distinct mechanisms. JAM-A participates in cell–cell adhesion and leukocyte transmigration, and disruption of JAM-A-associated barrier regulation may facilitate inflammatory cell recruitment under conditions of lung injury [58]. Importantly, primary studies have shown that neutrophil elastase can proteolytically cleave cell-surface γ-ENaC and increase ENaC-mediated Na^+^ current in heterologous and airway epithelial experimental systems [101]. This effect should be distinguished from the canonical furin cleavage site near γR143 [97,102]. More broadly, ENaC proteolytic regulation involves sequential processing of the extracellular domains of the α- and γ-subunits. Thus, neutrophil elastase should be considered within the broader framework of protease-dependent ENaC activation rather than as an isolated JAM-A–ENaC pathway [30]. Cingulin is a cytoplasmic tight junction-associated scaffold that interacts with junctional proteins and the cytoskeleton and can influence tight junction organization and paracellular ion permeability [59]. However, direct evidence that cingulin specifically controls ENaC vesicular trafficking or apical delivery is currently limited. Therefore, its potential relationship to ENaC is more appropriately considered indirect, through maintenance of epithelial polarity, cytoskeletal organization, and the junctional environment required for polarized membrane transport. Further experimental evidence is needed before a direct cingulin–ENaC trafficking mechanism can be established [103,104].

## 6. Conclusions and Perspectives

Current evidence strongly supports two complementary components of alveolar fluid homeostasis: ENaC-mediated apical Na^+^ entry drives vectorial sodium and fluid absorption, whereas epithelial tight junctions restrict paracellular ion and fluid back-leak and preserve transepithelial electrochemical gradients [2]. Disruption of either process can impair net alveolar fluid clearance, and their simultaneous dysfunction is likely to contribute substantially to pulmonary edema in ALI/ARDS [7]. Available studies support functional coupling between transcellular Na^+^ transport and paracellular barrier properties, but direct protein–protein regulatory pathways linking ENaC to individual tight junction proteins are supported less consistently [8,37]. In particular, proposed direct interactions involving ZO-1, occludin, JAM-A, cingulin, or specific claudin subtypes often derive from indirect evidence, non-alveolar epithelial systems, or experimental models that do not simultaneously assess ENaC function and barrier physiology [57,60]. However, this relationship is unlikely to be identical across all forms of ALI/ARDS. The heterogeneity should be considered when interpreting experimental findings and when selecting therapeutic targets [2].

Therapeutic strategies directed at ENaC and tight junction preservation remain at different stages of evidence. Mechanistic and cell-based studies demonstrate that ENaC expression, membrane abundance, and channel activity can be modified by hormonal signaling, intracellular pathways, and post-translational regulation [30,40]. Animal and other preclinical studies further suggest that enhancing epithelial sodium transport or preserving tight junction integrity may improve AFC and reduce pulmonary edema. However, direct ENaC-targeted treatment has not yet been established as an effective clinical therapy for ALI/ARDS [76,105]. Aldosterone-mediated stimulation of ENaC should currently be considered primarily a mechanistic or preclinical concept rather than a validated therapeutic intervention [49]. Systemic manipulation of ENaC activity may have important extra-pulmonary consequences because ENaC also plays a central role in renal sodium handling and systemic volume homeostasis [59]. Therefore, lung-selective modulation of ENaC would be preferable to non-specific systemic manipulation. Strategies targeting oxidative stress, inflammatory signaling, or tight junction preservation, including antioxidants and pharmacological stabilization of ZO-1, occludin, or claudins, are supported predominantly by experimental and preclinical evidence [13,45]. Statins have been evaluated clinically in ARDS, but large randomized trials have not demonstrated consistent improvement in major clinical outcomes [106].

Gene-based approaches aimed at restoring ENaC or tight junction function remain experimental and face substantial translational challenges, including cell-type-specific delivery, control of expression level, durability, immunogenicity, and safety [107]. Similarly, direct pharmacological targeting of tight junction proteins remains largely preclinical [108]. Overall, current evidence supports ENaC and tight junction regulation as biologically plausible therapeutic targets, but the translational gap remains substantial [8,76]. Future studies should prioritize validation in clinically relevant models, development of lung-selective delivery strategies, careful assessment of systemic adverse effects, and well-designed clinical trials before these approaches can be considered for routine ALI/ARDS treatment [2]. These integrated approaches will be essential for distinguishing direct molecular crosstalk from parallel responses to epithelial injury.

## Figures and Tables

**Table 1:** Major Tight Junction Proteins Involved in ALI/ARDS.

Protein Name	Physiological Function	Alterations in ALI/ARDS	Representative Regulatory Mechanisms/Factors	Model Systems	Potential Therapeutic implications	References
ZO-1	Links transmembrane proteins to the actin cytoskeleton	Reduced expression and redistribution from intercellular junctions to the cytoplasm	Inflammatory signaling, oxidative stress, phosphorylation, RhoA/ROCK-mediated cytoskeletal remodeling	In vitro epithelial cell models and mechanistic studies of ZO-1–actin organization	Preservation of junctional ZO-1 localization and stabilization of tight junction structure	[65]
Occludin	Maintains tight junction integrity and regulates paracellular permeability	Reduced expression, internalization, altered phosphorylation, and disrupted junctional localization	Src/PKC-dependent phosphorylation, oxidative stress, and protein degradation	Experimental epithelial models and human/experimental ARDS-related evidence	Prevention of occludin degradation and maintenance of junctional localization	[66]
Claudins	Determine paracellular ion selectivity and permeability	Subtype-dependent dysregulation, including reduced barrier-forming claudins such as claudin-4 and claudin-18	Inflammatory stimuli, oxidative stress, and altered epithelial signaling	Preclinical animal models of acute lung injury and epithelial barrier dysfunction	Restoration of barrier-forming claudins and stabilization of paracellular ion permeability	[37]
JAM-A	Supports cell–cell adhesion and regulates leukocyte transmigration	Reduced junctional abundance or increased shedding during inflammatory/mechanical injury	Mechanical stress and ADAM10/ADAM17-mediated shedding	Experimental epithelial/inflammatory models; mechanistic evidence of JAM-A loss or shedding	Preservation of JAM-A-mediated cell adhesion and limitation of excessive shedding	[82]

## Data Availability

No new data were created or analyzed in this study. Data sharing is not applicable to this article.

## Abbreviations

The following abbreviations are used in this manuscript:

AFC	Alveolar fluid clearance
ALI	Acute lung injury
ARDS	Acute respiratory distress syndrome
ENaC	Epithelial sodium channel
IL	Interleukin
JAM-A	Junctional adhesion molecule-A
LPS	Lipopolysaccharide
PDZ	Postsynaptic density-95/discs-large/zonula occludens-1
PKC	Protein kinase C
RhoA	Ras homolog family member A
ROCK	Rho-associated coiled-coil-containing protein kinase
ROS	Reactive oxygen species
TNF-α	Tumor necrosis factor-α
ZO-1	Zonula occludens-1
